# Heat and shear stability of particle stabilised foams for application in gluten-free bread

**DOI:** 10.1007/s13197-023-05794-0

**Published:** 2023-08-19

**Authors:** T. Schmid, R. Leue-Rüegg, N. Müller

**Affiliations:** Zurich University of Applied Science (ZHAW), Einsiedlerstrasse 34, 8820 Wädenswil, Switzerland

**Keywords:** Particle stabilised foams, Heat stability, Shear stability, Bakery, Gluten-free, Bread

## Abstract

Bread forms an integral part of the daily diet in many cultures worldwide. At the same time, a significant number of people try to avoid wheat-based products for either health reasons or due to personal preferences. The absence of a protein network in gluten free bread affects its structure, taste, texture and shelf-life. This paper suggests a technological solution to this issue that uses a pre-foamed mass of gluten free raw materials which is mixed with the bread’s ingredients, then kneaded and baked to form a high quality gluten free bread. To survive the high shear stresses during kneading and temperature increase during baking, the foam requires exceptional stability. This stability was achieved through particle stabilisation of the bubble interfaces. Both of the tested foams (with and without particles) exhibited thermal stability up to 80 °C. However, resistance to shear stresses was higher in the particle stabilised foams. Of all the tested particles, linseed press cake and banana powder led to the best results. In conclusion, particle stabilised foams seem very well suited to applications in gluten free baked goods. Further application potential is seen for vegan foamed desserts.

## Introduction

The structure and texture of regular wheat bread is heavily dependent on the gluten network formed during the dough-making process, which is then stabilised during baking. The absence of a protein network in gluten free material affects the formation of pores that are typically found in the bread crumb; both the number and size of pores is reduced. Additives such as emulsifiers, stabilisers, oil or egg are often added to minimise this effect, resulting in a longer list of ingredients and, in many cases, a poorer nutritional profile (Allen and Orfila 2018).

An alternative path to achieving the desired bread structure and texture is through an adaptation to the processing protocol. Existing approaches include the extrusion of gluten-free bread dough to introduce and expand gas in the loaf (Lammers et al. 2018), a change in viscoelastic properties of the dough through high pressure processing (Vallons et al. 2011) or rapid structure stabilisation through Ohmic heating (Bender and Schönlechner 2020).

In this work, an alternative technological approach was tested in which a pre-foamed mass was produced from ingredients suited for gluten-free breads. This foam was then mixed with the rest of the ingredients, kneaded and baked to form a high-quality gluten free bread. To survive the harsh treatment of the bread making process, i.e. the high shear stresses from kneading and the heat of baking, a foam requires exceptional stability. This stability was achieved through particle stabilisation of the bubble interfaces. Finely milled linseed presscake, banana powder and microcrystalline cellulose were chosen as the particles as work by Rüegg et al. (2022) showed their superiority in foam stabilisation.

Classic low molecular weight emulsifiers decrease interfacial tension, provide stability through steric hindrance, sometimes coupled with electrostatic repulsion, and hence result in fast bubble stabilisation (Rayner et al. 2014). Stabilisation of particles with partial dual wettability is slower, nevertheless the adsorption of particles at the interface is considered extremely stable if not irreversible (Dickinson 2010; Gonzenbach et al. 2006). Particle stabilised foams have been receiving increased attention in the last decades as long-term stability of aerated products is more difficult to achieve than for emulsions (Hunter et al. 2008; Vignes-Adler and Weire 2008). Dickinson et al. (2012) highlighted the importance of surface wettability (contact angle), particle concentration, shape and size of the particles. Tavernier et al. (Tavernier et al. 2016) assembled an overview of food-grade particles with the potential to serve as pickering particles, such as fats and wax crystals, protein-polysaccharide complexes, flavanoids and protein particles (Rayner et al. 2014). Additional food grade particles of interest described in the literature include chitin nanocrystals (Belamie et al. 2004), soy protein aggregates (Liu and Tang 2013) and hydrophobins produced by filamentous fungi that exhibit rigidness similar to small particles (Linder 2009).

The most recent review articles from Amani et al. (2022), and Linke and Drusch (Linke and Drusch 2018) provide a detailed discussion of the opportunities and limitations of pickering type emulsions and foams in food applications, emphasizing the fact that systems with a high internal phase volume, as is typical in foamed systems, can be stabilised by applying a small quantity of particles. While moderately hydrophobic particles can generally stabilise foams well, they do not always sufficiently support the formation of the foam due to slower adsorption at the interface than low molecular surfactants. Three particle-emulsifier interactions are known, i.e. (i) a decrease in interfacial tension to values below the effect of a pure surfactant when particles are added to the surfactant, (ii) an increase in particle contact angle due to the addition of a surfactant and (iii) the promotion of particle flocculation, which affects the geometrical structure at the interface, and is observable through changes in the interfacial rheology (Hunter et al. 2008).

This investigation is the first to examine shear and thermal stability of foams that have been stabilised with a combination of emulsifiers and particles. These characteristics are crucial for foams that are subject to heat and shear stresses during further processing into food products such as gluten-free bread.

## Materials and methods

### Preparation of particles

Particles from linseed presscake (Bio quality, Goldmühle, Altstätten, CH) and banana powder (Spiceworld GmbH, Salzburg, Austria) were cooled in liquid nitrogen and pre-milled in a Thermomix (Vorwerk, Dierikon, CH), before being micromilled on a three roller mill (hydraulic three roller mill, type SDY 200, Bühler, Uzwil, CH) at 20 °C and 10–12 bar. Microcrystalline cellulose (type VIVAPUR®, E460, JRS Pharma Family, Patterson, USA) was used without any further milling. Tab. 1 lists the properties of the different particles according to Rüegg et al. (2022) (with permission). The mean particle size and particle distribution width plus particle shape were measured by means of a microscopic analysis by an external, accredited laboratory. The wetting angle was analyzed by pressing particles into dense tablets (n = 6) using a tablet press (MTQX-1, GlobePharma, USA), adding a defined droplet of water to the surface and quantifying the contact angle (Contact Angle System OCA Goniometer, dataphysics, Germany). For linseed presscake and banana powder the listed characteristics are after milling.

**Table 1:** Particle characteristics of micromilled banana powder, micromilled linseed press cake and microcrystalline cellulose (Rüegg et al. 2022, with permission)

	Median particle size [µm]	Particle shape [–]	Wetting angle [°]
Banana powder	5.5	Roundish	53.1 ± 1.6
Linseed press cake	2.9	Roundish	74.8 ± 1.6
Microcrystalline cellulose	2.8	Elongated	27.5 ± 4.0

### Mix preparation

8460 g (93%) of water was heated to 85 °C using an IKA heating rod, then 90 g (1.0%) of the emulsifier polyglycerol ester (PGE) was added to half of the water and 270 g (3.0%) potato starch (Fécule, Pistor, Rothenburg, CH) to the other half of the water. The latter mixture was stirred using a Polytron (Chemcol, Ytron Process Technology GmbH & Co. KG, Bad Endorf, Germany) for 1.5 min at a shear rate of 15,695 1/s. Both mixtures were then held at 85 °C for 10 min and cooled down to 4 °C. As soon as the PGE-water mixture reached 45 °C, 270 g (3.0%) goldpea protein (Golden Pea Protein, Alver, Saint Aubin, CH) was added and homogenized at 15,695 1/s for 30 s using the Polytron. Thereafter, the starch–water mixture and the PGE-protein-water mixture were combined and homogenized for 1 min at 15,695 1/s using the Polytron. Finally, the mixture was stored at 4 °C overnight. On the next day and directly before foaming, particles were added to the mix to reach a final particle concentration of 3.0% before being mixed again at 8561 1/s for 1.5 min.

### Foaming

Foaming was performed in a rotor–stator whipping device (model MT-FM50, Kinematica AG, Malters, CH) with a 50/6 geometry at a shear rate of 4922 1/s and a gas fraction of 0.5. The mix and air flow rates were adjusted to reach a total throughput rate of 36 l/h for all trials. The machine head was cooled with tap water to keep the foam temperature between 20 and 25 °C for all trials. All foaming trials were repeated six times.

### Analysis

All analyses were performed immediately after foaming, and after 1, 4 and 12 days of storage at 4 °C. Foams were stored in plastic containers of 144.3 ± 0.5 ml with a screw top.

### Gas volume fraction

The achieved gas volume fraction θ_V_ relating the volume of gas to the total volume of the foam was measured based on the product density before (ρ_liquid_) and after foaming (ρ_foam_) using the following formula:

1 $${\varnothing }_{V }[-]=\frac{\frac{OR [\%]}{100}}{\frac{OR [\%]}{100}+1}$$

where overrun (OR):

2 $$OR [\%]=\frac{{\rho }_{liquid} \left[\frac{\mathrm{kg}}{\mathrm{m}3}\right]-{\rho }_{foam } \left[\frac{\mathrm{kg}}{\mathrm{m}3}\right]}{{\rho }_{foam} \left[\frac{\mathrm{kg}}{\mathrm{m}3}\right]}\cdot 100\mathrm{ \%}$$

Gas fractions were determined in triplicate.

### Bubble size distributions

Micrographs were obtained using an inverted light microscope (Revolve RLV-100-G, Discover, Echo Inc., San Diego, USA) using an objective with 20× magnification in order to capture a minimum of 50 bubbles and a maximum of 1000 bubbles per image. Image analysis was performed using BubbleAnalyser© (ZHAW internal software, validated against BubbleDetect© (Copyright 2003 Lab of Food Process Engineering (LMVT), ETH Zurich, Switzerland, Müller-Fischer et al. 2007), with the image binarized using a dynamic threshold set at 15% darkest pixels followed by a contour finding algorithm. Cumulative number distribution functions were then derived from the resulting contour diameters and the median bubble diameter x_50,0_ and distribution width x_90,0_/x_10,0_ were also determined. Six micrographs were taken for each foam to achieve a total number of values per parameter setting of n = 36.

### Drainage

Drainage was measured by filling 120 ml cups marked with a volume scale. Drained material was noted directly after filling (0 day) and at 1 day, 4 days and 12 days after foaming. The percentages were then converted into drained volume per original foam volume. Drainage was determined in triplicate to achieve a total number of values of n = 18.

### Heat stability

A small layer of foam was carefully transferred to an object slide, then inserted into the temperature control system (stage model T96-P, Linkam Scientific Instruments LTD, Surray, UK) and observed under an inverse light microscope (Revolve RLV-100-G, Discover, Echo Inc., San Diego, USA) as it was heated from 20 to 110 °C at a heating rate of 10 °C/min. Images were taken at 20, 30, 40, 50, 60 70, 80, 90, 100 and 110 °C. Each foam was produced 6 times, then analysed for its heat stability to achieve a total number of data points of 6 per foam.

### Shear stability

Each foam was produced six times, then poured into a rheometer (model MCR 702 multidrive, Anton Paar Switzerland AG, Buchs, CH) cylinder with a CC27/T200/AL geometry and sheared at the following shear rates: 1, 100, 500 and 1000 1/s (each shear rate was maintained for 60 s), and then at 3000 1/s for 480 s. Viscosity was measured throughout the shearing process (results not shown). Afterwards, the foam was removed from the cylinder and 6 microscope images were taken for each foam to assess the bubble size distribution microscopically (n = 36).

### Statistical analysis

For statistical analysis, bubble size distribution data was subjected to a Kruskal Wallis test (a = 0.05) followed by an unpaired Wilcox test using RStudio statistical software (Version 1.4.1717, RStudio PBC, Boston MA, US). The results with significant differences are indicated by different letters in the graphs.

## Results and discussion

**Fig. 1:**
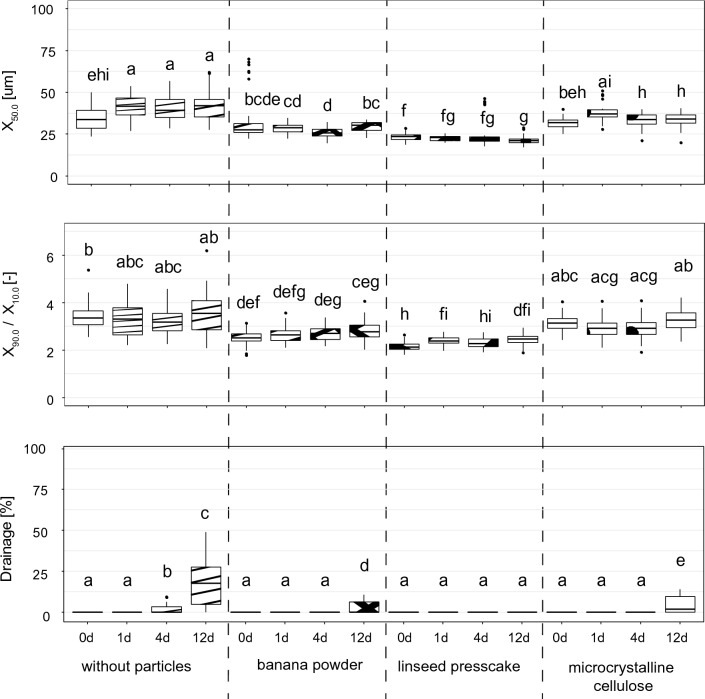
Median bubble sizes (top, n = 36), bubble size distribution width (middle, n = 36) and drainage (bottom, n = 18) of foam produced with the rotor–stator device, geometry 50/6 at a gas fraction of 0.5 and a shear rate of 4922 1/s measured directly after foaming (0d) and after 1,4 and 12 days of storage. Foams were produced without particles (left), with banana powder (second from left), with linseed presscake (third from left) and with microcrystalline cellulose (right). Letters (a, b, …) indicate classes of significance based on the analysis using a Kruskal–Wallis test (α = 0.05) followed by an unpaired Wilcoxon test using statistical software (RStudio V 1.4 1717, Boston MA, US)

Figure 1 shows the median bubble size X_50,0_, the bubble size distribution width X_90,0 /_ X_10,0_ and the drainage of fresh foams that were produced without particles or with banana powder, linseed presscake or microcrystalline cellulose after 0, 1, 4 and 12 days of storage. The results show that at each point in time, the median bubble sizes of the soy protein aggregate foams produced with banana powder or linseed presscake were significantly smaller than those from foams produced without particles. The only exception was banana powder at time 0. Furthermore, the median bubble sizes for the foams produced with linseed presscake were significantly smaller than for banana powder. For the foams produced with microcrystalline cellulose, the bubble sizes at time 0 and 1 were not significantly different to foams without particles but contained significantly smaller median bubble sizes after 4 and 12 days of storage, suggesting the enhanced long-term stability of these foams. In terms of bubble size distribution widths, the trends were similar, i.e. foams with banana powder showed significantly narrower bubble size distributions than foams without particles. Stabilisation through the addition of linseed presscake led to the narrowest bubble size distributions, while foams with microcrystalline cellulose were comparable to foams without particles. Drainage rates were low for all samples and did not significantly change during the first day for particle free foams and over 4 days for foams with banana powder and microcrystalline cellulose. The foams stabilised with linseed presscake, however, remained completely stable for 12 days.

The smaller median bubble diameter of foams stabilised with linseed press cake and banana powder versus the pure stabilisation using the PGE emulsifier can be attributed to dosage dependent optimisation of the stabilisation effect for emulsifier-particle systems. Low dosages of emulsifier (in the range used in this work) are known to reduce surface tension and increase foam stability while a further increase in emulsifier dosage is known to lead to competition between the emulsifier and the particles (Hunter et al. 2008; Midmore 1998). Previous work using a model recipe with milk protein and guar found similar tendencies for comparable processing conditions, i.e. a slight reduction in median bubble size upon addition of particles (Rüegg et al. 2022).

The differences in the median bubble sizes of the foams with microcrystalline cellulose versus the banana powder and linseed press cake foams cannot be correlated with particle size, as the particle size of microcrystalline cellulose (2.8 µm) was in the same range as linseed presscake (2.9 µm) and smaller than banana powder (5.5 µm). According to Kaptay, an upper limit in particle size of 3 µm should not be exceeded, since foam stabilisation would be ineffective above this value (Kaptay 2004). Nevertheless, this seems not to be the case for banana powder. A further point to consider is that the elongated particle shape of the microcrystalline cellulose might not have resulted in optimal surface coverage during foaming, unlike the rounded shape of both linseed press cake and banana powder. Furthermore, the very low wetting angle of microcrystalline cellulose of 27.5 ± 4.0° is also seen as critical. While Fameau and Salonen (2014) and Stocco et al. (2011) observed that wetting angles close to but below 90° are preferred, even more detailed studies by Schwarz (s) and Ata et al. (2002) found that particle contact angles of around 63° and 66° were optimal for foam stabilisation. This observation further explains the slight yet significant increase in stability of the foams stabilised with linseed press cake (contact angle 74.8 ± 1.6°) over banana powder (contact angle 53.1° ± 1.6°).

**Fig. 2:**
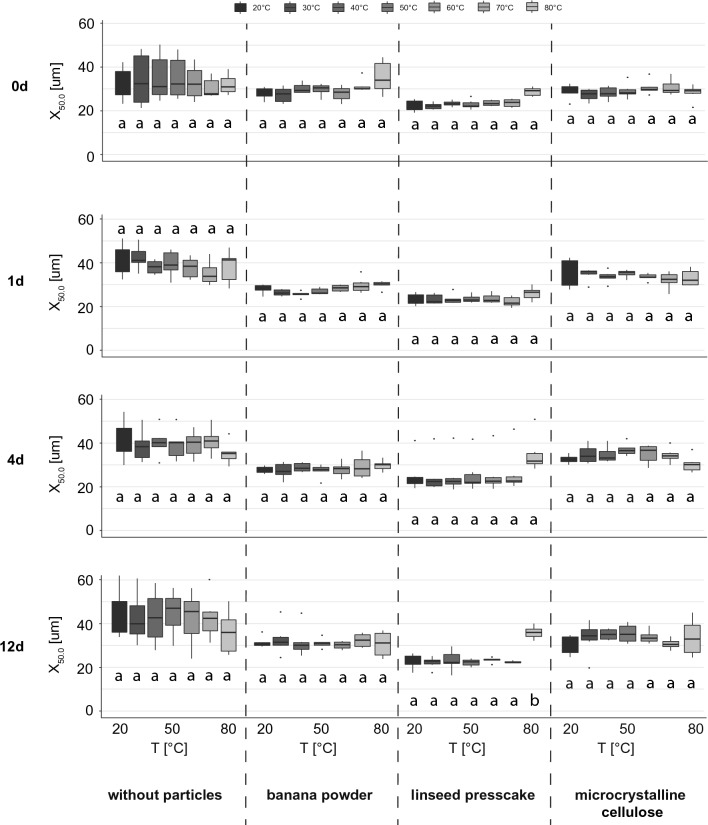
Change in median bubble size viewed under a microscope during heating of the foams from 20 to 80 °C (n = 6). The foams were produced in a rotor–stator device, geometry 50/6 at a gas fraction of 0.5 and a shear rate of 4922 1/s. Measurements were taken directly after foaming (0d) as well as after 1, 4 and 12 days storage time. The foams were produced without particles (left), with banana powder (second from left), with linseed presscake (third from left) and with microcrystalline cellulose (right). Microscope images were taken at 20, 30, 40, 50, 60, 70 and 80 °C and median bubble sizes determined

**Fig. 3:**
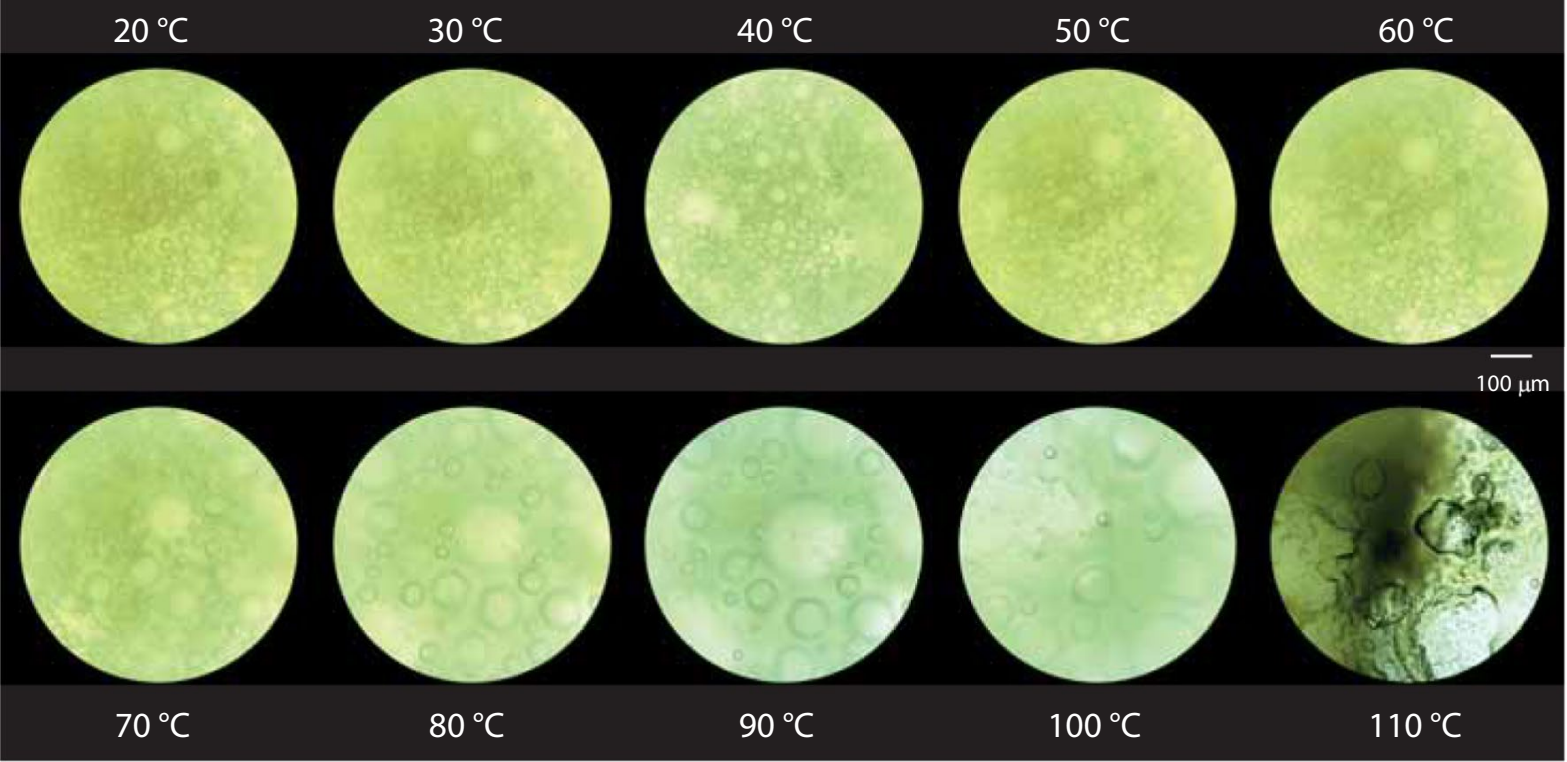
Light microscope images of foam during heating from 20 to 110 °C measured using an objective with 20 × magnification. The exemplarily foams shown contain linseed presscake particles and were analysed 12 days after production. The foams were produced using a rotor–stator device, geometry 50/6 at a gas fraction of 0.5 and a shear rate of 4922 1/s

Figure 2 shows the median diameters of foams produced with and without the addition of particles. They were either directly heated to 110 °C while the effect on the bubble sizes was assessed or stored for 1, 4 or 12 days before the foams’ thermal stability was tested. Figure 3 shows micrographs taken at each temperature interval up to 110 °C for the foam containing linseed press cake.

When assessing thermal stability through the evaluation of median diameters, all foams remained stable up to 80 °C. The median bubble diameter increased by less than 50% when heated to 80 °C. Only for 12-day old foams containing linseed presscake was a significant change in median diameter observed upon heating from 70 to 80 °C. At temperatures above 80 °C all of the foams (with and without particles) changed their structure to an extent where it was not possible to determine median diameters.

For the foams without particles, the ranges of the resulting median diameters were wide and, thus, the reproducibility lower than for the foams with particles, i.e. the difference from smallest to largest median diameter was higher for foams that did not contain particles. The underlying reason is with high probability due to the weaker interphase stabilisation of bubbles known from purely emulsifier stabilised foams compared to foams containing particles (Dickinson 2010; Gonzenbach et al. 2006). The resulting large deviation in foam quality between different repetitions is seen as critical when applying the procedure to products such as gluten free bread, since consistent product quality is extremely important. Hence, the addition of particles is recommended to ensure good reproducibility of the results.

The observed high thermal stability of the foams shows promise for future baking applications since starch gelatinisation starts at around 55 °C and is complete at about 80 °C for most starches, with the exception of some millets, for which gelatinisation does not finish until temperatures close to 90 °C (Ubwa et al. 2012). As a result, it can be assumed that gelatinised starch is predominantly responsible for the structure stabilisation in gluten free bread at high temperatures, and foam stability is of secondary importance.

In food applications, good thermal stability of foams can be seen in products such as meringue and angel food cake. In such systems, bubble sizes and cake volume increase greatly up to temperatures of 70 °C which is followed by strong structural stabilisation at temperatures above 70 °C through the combined effect of protein denaturation and starch gelatinisation (Foegeding et al. 2006). Thermal stability of particle stabilised foams containing emulsifiers has so far only been discussed for non-food applications such as firefighting foams (Sheng et al. 2021), thermal insulation (Huang et al. 2018) and ceramic foams (Gonzenbach et al. 2006). In most of these applications, even higher thermal stability was achieved through the use of non-food grade nanoparticles with adapted hydrophobicity. However, in this study food-grade particles in the low micrometer range were examined.

**Fig. 4:**
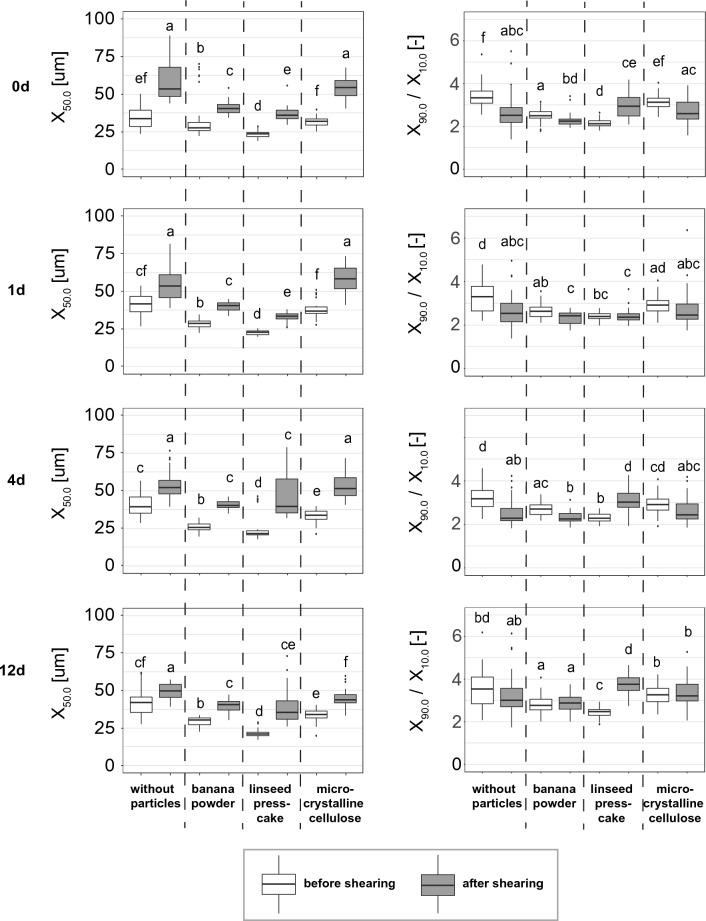
Median bubble sizes (left, n = 36) and bubble size distribution width (right, n = 36) before and after shear testing of freshly produced (0d) and 1, 4 and 12 day old foams, both with and without particles, produced with a rotor–stator device, geometry 50/6, a gas fraction of 0.5 and a shear rate of 4922 1/s. Shear stress was adapted to mimic dough kneading and the following shear rates were applied consecutively: 1, 100, 500 and 1000 1/s (each shear rate was maintained for 60 s), then at 3000 1/s for 480 s. Letters (a, b, …) indicate classes of significance based on the analysis by a Kruskal–Wallis test (α = 0.05) followed by an unpaired Wilcoxon test using statistical software (RStudio V 1.4 1717, Boston MA, US)

Median bubble diameters as well as the distribution widths before and after shearing at 1, 100, 500 and 1000 1/s (each shear rate maintained for 60 s), then at 3000 1/s (for 480 s) are shown in Fig. 4 to mimic the foams’ ability to survive shear at conditions that are similar to the shear that a dough experiences during the kneading process in bread production. The results show that all foams survived the shear treatment and were only moderately changed. The median bubble sizes increased significantly for all foams, but in ranges that are acceptable for the intended application in gluten free doughs. The absolute increase in median bubble diameter was the lowest for foams stabilised with linseed press cake (increase by 12.4 µm from 23.5 to 35.9 µm) and banana powder (increase of 12.8 µm from 27.6 to 40.4 µm). The foam stabilized with microcrystalline cellulose and the one without particles, however, exhibited increases in median bubble diameters of 22.5 and 19.9 µm, respectively. The relative increase was 52.9% for linseed press cake, 46.0% for banana powder, 70.3% for microcrystalline cellulose and 59.1% for foams without particles. The bubble size distribution width changed significantly for most of the foams, rising for foams with linseed presscake at d0, d4, d12 and falling for all of the others. No clear trend can be derived from the change in distribution width.

The rheology of foams has been investigated in detail in order to predict foam behaviour in flow. In most cases, liquid food foams like all of the foams investigated in this study exhibit a shear-thinning behaviour (e.g. Laporte et al. 2015; Müller-Fischer et al. 2008). The shear-thinning behaviour is caused, among other things, by an elongation and alignment of bubbles when exposed to shear forces. This is why the high foam stability (Fig. 4) observed for both the foams with and without particles was to be expected. Upon investigating the effect of different additives on foam stability in 3D printing, Lee et al. (2021) found that the addition of xanthan gum to both egg white and vegan foams led to excellent foam stability even allowing complex shapes to be printed. The underlying effect was attributed to the shear thinning behaviour of xanthan gum, which optimally supported the foam structure when exposed to high shear forces in the 3D printing nozzle.

The further increase in foam bubble stability when particles are added might be explained by a phenomenon found by Wang and Brito-Parada (2022): The dynamics of collisions between particle-laden bubbles and the air–liquid interfaces were observed in order to understand the enhanced stability of particle stabilised foams. The authors found a damping effect of particle laden bubbles when there were collisions caused by a movement of the particles over the bubble surface. This effect was found to be greater as particle coverage increased.

This increase in foam stability against both thermal and mechanical stresses is expected to improve the quality of gluten-free bread as such a foam is sufficiently stable to survive both the kneading and the baking process and, thus, elevates the number of gas pores in the final gluten-free bread.

## Conclusion

In this study, the time-, shear- and thermal stability of particle stabilised foams was assessed and compared to a purely emulsifier stabilised reference foam. It can be concluded that the addition of particles led to significantly enhanced stability that reduced drainage and bubble growth over time. The same was found to be true when shear forces were applied to the samples. Within the tested particles, finely milled linseed press cake and banana powder led to better foam quality and stability than microcrystalline cellulose. This was attributed to the differences in contact angle and particle shapes. Further fundamental work to understand the underlying mechanisms of stabilisation in more detail should be performed in the future to transfer the findings to other recipes.

In contrast, the addition of particles was found to have no significant effect on thermal stability, with even the reference foams without particles remaining stable up to 80 °C. However, the reproducibility of the results of the thermal stability tests was improved in the particle stabilised systems.

Overall it can be concluded that the particle stabilised foams tested are extremely promising for applications in gluten free bread. Furthermore, particle stabilised foams could also be used in vegan desserts such as vegan meringue or mousse products.

## Data Availability

The datasets used and/or analysed during the current study are available from the corresponding author on reasonable request.

## Abbreviations

d: Days
PGE: Polyglycerol ester
